# Unexpected positive control of NFκB and miR-155 by DGKα and ζ ensures effector and memory CD8^+^ T cell differentiation

**DOI:** 10.18632/oncotarget.8164

**Published:** 2016-03-17

**Authors:** Jialong Yang, Ping Zhang, Sruti Krishna, Jinli Wang, Xingguang Lin, Hongxiang Huang, Danli Xie, Balachandra Gorentla, Rick Huang, Jimin Gao, Qi-Jing Li, Xiao-Ping Zhong

**Affiliations:** ^1^ Department of Pediatrics, Division of Allergy and Immunology, Duke University Medical Center, Durham, NC, USA; ^2^ School of Laboratory Medicine, Wenzhou Medical University, Wenzhou, Zhejiang, China; ^3^ Department of Oncology, Nanfang Hospital, Southern Medical University, Guangzhou, Guangdong, China; ^4^ Department of Immunology, Duke University Medical Center, Durham, NC, USA

**Keywords:** DGK, miR-155, NFκB, CD8 T cell, Listeria monocytogenes, Immunology and Microbiology Section, Immune response, Immunity

## Abstract

Signals from the T-cell receptor (TCR) and γ-chain cytokine receptors play crucial roles in initiating activation and effector/memory differentiation of CD8 T-cells. We report here that simultaneous deletion of both diacylglycerol kinase (DGK) α and ζ (DKO) severely impaired expansion of CD8 effector T cells and formation of memory CD8 T-cells after *Listeria monocytogenes* infection. Moreover, ablation of both DGKα and ζ in preformed memory CD8 T-cells triggered death and impaired homeostatic proliferation of these cells. DKO CD8 T-cells were impaired in priming due to decreased expression of chemokine receptors and migration to the draining lymph nodes. Moreover, DKO CD8 T-cells were unexpectedly defective in NFκB-mediated miR-155 transcript, leading to excessive SOCS1 expression and impaired γ-chain cytokine signaling. Our data identified a DGK-NFκB-miR-155-SOCS1 axis that bridges TCR and γ-chain cytokine signaling for robust CD8 T-cell primary and memory responses to bacterial infection.

## INTRODUCTION

CD8 T cells are critical for controlling and eradicating intracellular pathogens and tumors. Naïve CD8 T cells are activated, massively expand, and differentiate into cytotoxic, inflammatory cytokine-secreting effector T cells after encountering antigen-presenting cells loaded with pathogen-derived antigenic peptides in their MHC class I molecules. Engagement of the TCR on naïve CD8 T cells provides a critical signal that initiate their activation and expansion. At the peak of CD8 T cell responses, effector CD8 T cells contain short-lived effector cells (SLECs, CD127^low^KLRG1^hi^) and memory precursor effector cells (MPECs, CD127^hi^KLRG1^low^) [1, 2]. SLECs are able to produce high levels of cytokines but are poised to die, while MPECs have high potential to differentiate to long-lived memory cells. Following clearance of infecting pathogens, effector CD8 T cells undergo precipitous contraction, with the majority of pathogen-specific effector CD8 T cells dying by apoptosis; but typically a small percentage (~5%-10%) of these cells survive to become memory cells [3]. Memory T cells are the foundation of both adaptive immunity and vaccination.

Both TCR signal strengths and quality have been demonstrated to regulate not only the magnitude but also the effector/lineage fates of responding CD8 T cells [4–7]. An important event following TCR engagement is the activation of phospholipase Cγ1, which hydrolyzes phosphatidylinositol bis-4,5-phosphate (PIP2) to produce two important second messengers, diacylglycerol (DAG) and inositol 1,4,5-trisphosphate (IP3). DAG associates with and allosterically activates RasGRP1 and PKCθ, leading to the activation of the Ras-Erk1/2-AP1 and CARMA1/Bcl10/MALT-IKK-NFκB signaling pathways that are indispensable for T cell activation and effector/memory CD8 T cell differentiation [8–11]. Additionally, the Ras-Mek1/2-Erk1/2 pathway and CARMA1 mediate TCR-induced mTOR activation [12, 13]. mTOR and its tight regulation by TSC1/2 have also been found to control effector/memory CD8 T cell fate decisions [14–18]. In addition to TCR, γ-chain cytokine receptors such as IL-7 receptor, IL-2 receptor, and IL-15 receptor are important determinants of CD8 effector/memory differentiation and memory T cell homeostasis [2, 19, 20]. Interestingly, microRNA (miR)-155 controls both effector CD8 T cell expansion and memory CD8 T cell formation at least in part by promoting γ-chain cytokine signaling *via* targeting SOCS1, a negative regulator of γ-chain cytokine receptor signaling [21]. Whether and how TCR signaling and γ-chain cytokine signaling cross-regulate has been unclear.

DAG kinases (DGKs) are a family of 10 enzymes that catalyze phosphorylation of DAG into phosphatidic acid (PA) and thus inhibit DAG-mediated signaling in mammals [10, 22]. DGKα and ζ are the major isoforms expressed in T cells [23–25]. Previous studies have demonstrated that both isoforms are involved in negative controls of T cell activation [23–27]. Deficiency of either DGKα or ζ resulted in enhanced effector CD8 T cell expansion but slightly decreased memory CD8 T cell responses to lymphocytic choriomeningitis virus (LCMV) infection [27, 28]. However, these studies were performed in germline knockout mice, and thus CD8 T cell extrinsic factors could not be completely ruled out. Additionally, whether these two isoforms may function redundantly or synergistically to control CD8 T cell effector/memory responses is unclear.

In this report, we utilized a newly generated, DGKζ-conditional deficient mouse model in combination with DGKα germline-deficient mice, the OT1 TCR transgenic model, and the model of *Listeria monocytogenes* that expresses ovalbumin (*LM-OVA*) [29]. We demonstrated that simultaneous ablation of both DGKα and ζ (DKO) in naïve CD8 T cells resulted in severe impairment of expansion and functioning of effector CD8 T cells during primary responses to *LM-OVA* infection due to impaired recruitment to and priming in draining lymph nodes (dLNs). Additionally, DKO hindered memory CD8 T cell formation and jeopardized maintenance of these cells due to increased death and reduced homeostatic proliferation. Although DKO CD8 T cells displayed elevated NFκB activation in steady state, they were impaired in TCR-induced NFκB activation in CD8 T cells, which led to decreased miR-155 expression, subsequent increased SOCS1 expression, and impaired γ-chain cytokine signaling. Reconstitution of miR-155 expression in DKO OT1 T cells fully restored the cells' effector response and memory formation/maintenance. Thus, DGKα and ζ function as pivotal controllers during TCR signaling to ensure NFκB-induced miR-155 expression to target SOCS1 for subsequent γ-chain cytokine signaling in CD8 T cells.

## RESULTS

### Deficiency of both DGKα and ζ impairs effector and memory CD8 T cell differentiation

We previously used DGKα or DGKζ germline knockout (DGKαKO or DGKζKO) mice and demonstrated that a deficiency of either DGKα or DGKζ enhanced effector CD8 T cell expansion after viral infection [28]. Using DGKαKO and DGKζKO mice carrying the OT1 TCR transgene, which encodes a TCR-recognizing ovalbumin peptide_257-264_ (SIINFEKL) presented by H2K^b^ and thus directing T cell development to the CD8 lineage [30], we also found that a deficiency of either DGKα or ζ caused enhanced expansion of OT1 T cells following infection with *LM-OVA* (data not shown). To determine whether DGKα and ζ play a redundant or synergistic role during CD8 T cell-mediated immune responses, we generated DGKα^−/−^ζ*^f^*^/^*^f^*-ERCre (DKO) mice carrying the OT1 transgene so that both DGKα and ζ could be ablated following tamoxifen-induced deletion of DGKζ (manuscript in preparation). DKO OT1 T cells displayed slight increases of CD44^+^CD62L^−^ and CD44^+^CD62L^+^ effector/memory populations (Figure S1a). However, DKO CD44^−^CD62L^+^ naïve OT1 T cells expressed similar levels of CD25, CD69, CD122, T-bet, and Eomesodermin (Eomes), except with slightly upregulated CD127, a component of the IL-7 receptor, compared with wild-type (WT) OT1 T cells (Figure S1b).

**Figure 1:**
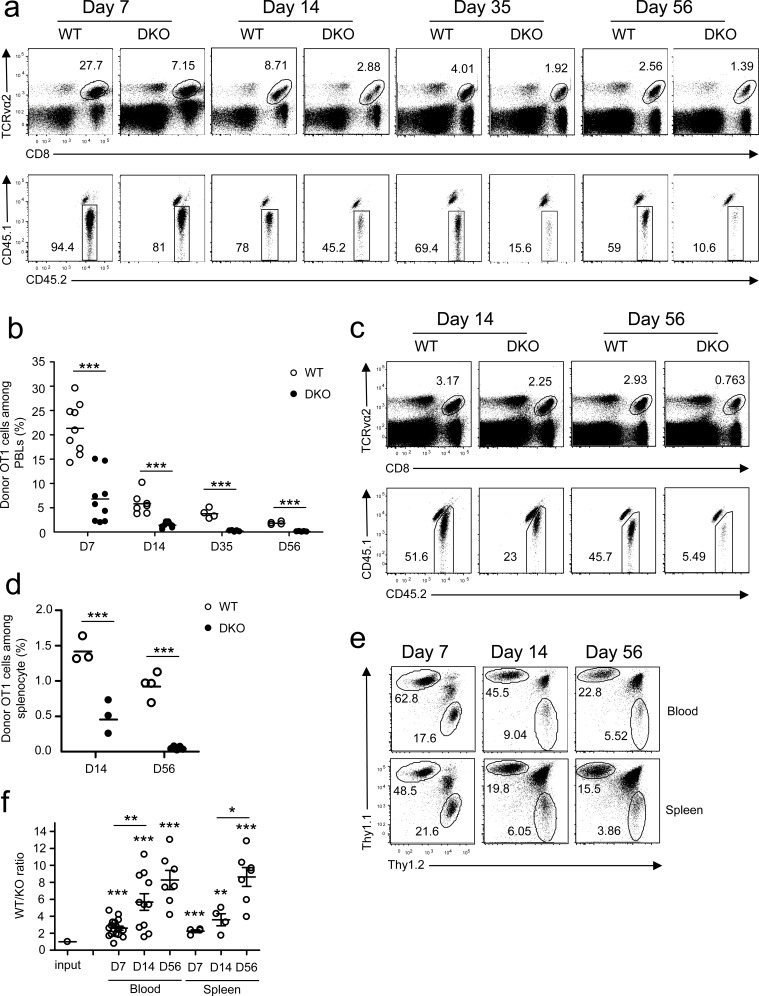
Deficiency of both DGKα and ζ impaired CD8 T cell responses **a.**-**d.** CD45.1^+^CD45.2^+^ congenic mice *iv* injected with 1 × 10^4^ CD45.2^+^Vα2^+^CD8^+^ WT or DKO naïve OT1 T cells were infected with *LM-OVA* on day 0 and examined on indicated days later. **a.** Representative dot plots of RBC-depleted peripheral blood leukocytes (PBLs). Top panels: CD8 and TCRVα2 staining of PBLs. Bottom panels: Donor-derived CD45.1^−^CD45.2^+^ OT1 cells from the gated TCRVα2^+^CD8^+^ population. **b.** Percentages of OT1 T cells in PBLs. Bars represent mean ± SEM. **c.** Representative dot plots of splenocytes. **d.** Percentages of donor-derived OT1 T cells in splenocytes. **e.**-**f.** Thy1.1^+^Thy1.2^+^ congenic mice *iv* injected with a mixture of 5 × 10^3^ Thy1.1^+^ WT and 5 × 10^3^ Thy1.2^+^ DKO naïve OT1 T cells were infected with *LM-OVA* and analyzed similarly to the method described in **a.**-**d. e.** Representative dot plots of Thy1.1 and Thy1.2 staining in gated TCRVα2^+^ PBLs and splenocytes. **f.** WT to DKO OT1 ratios in blood and spleen from individual mice. Data shown are representative of two independent experiments. Each circle represents one recipient mouse injected with WT and/or DKO OT1 T cells. *, *P* < 0.05; **, *P* < 0.01; ***, *P* < 0.001 (Student's *t* test).

To examine how DGKα and ζ double deficiency affected CD8 T cell responses *in vivo*, we adoptively transferred CD45.2^+^ naïve OT1 T cells from WT OT1 or DKO OT1 donors into CD45.1^+^CD45.2^+^ congenic recipient mice and infected the recipients with *LM-OVA*. In contrast to robust expansion of WT OT1 T cells detected in the peripheral blood, DKO OT1 T cell expansion was blunted on day 7 after infection (Figure 1a-1b), revealed by decreased CD8^+^TCRVα2^+^ cells and decreased donor-derived CD45.1^−^CD45.2^+^ OT1 T cells within these CD8^+^TCRVα2^+^ cells. Moreover, memory CD8 OT1 T cells were also severely decreased in recipients with DKO OT1 T cells 35 and 56 days after infection. Severe reduction of DKO OT1 effector and memory T cells was also found in the spleen of recipients (Figure 1c-1d). Recent studies have found that substantial numbers of CD8 T cells migrate to non-lymphoid organs during immune responses to form tissue resident memory cells [31]. The decreases of DKO OT1 T cells were not limited to peripheral blood, as they were also markedly decreased in peripheral lymph nodes, mesenteric lymph nodes, Peyer's patches, liver, lung, intestines and bone marrow (Figure S2). Thus, initial expansion and memory formation in primary response to a bacterial pathogen by DKO CD8 T cells were severely compromised.

To exclude possible contributions of differences in antigen clearance between WT and DKO OT1 T cell recipients to the blunted response of DKO OT1 T cells, we co-transferred both Thy1.1^+^ WT and Thy1.2^+^ DKO naïve OT1 T cells into Thy1.1^+^Thy1.2^+^ congenic recipients at a 1:1 ratio. Days 7, 14, and 56 after *LM-OVA* infection, DKO OT1 frequencies were substantially lower than in WT controls (Figure 1e), leading to increased WT to DKO ratios in both peripheral blood and spleen in individual recipients (Figure 1f). Moreover, WT/DKO ratios progressively increased from days 7 to 56, suggesting that DKO OT1 T cells might also be impaired in memory formation/maintenance.

Thus, although deficiency of either DGKα or ζ enhanced primary CD8 T cell responses to viral and bacterial infection, simultaneous loss of DGKα and ζ severely impaired CD8 cell expansion and memory formation.

### Loss of DGKα and ζ skewed CD8 T cell effector/memory programs and crippled CD8 T cell effector function

**Figure 2:**
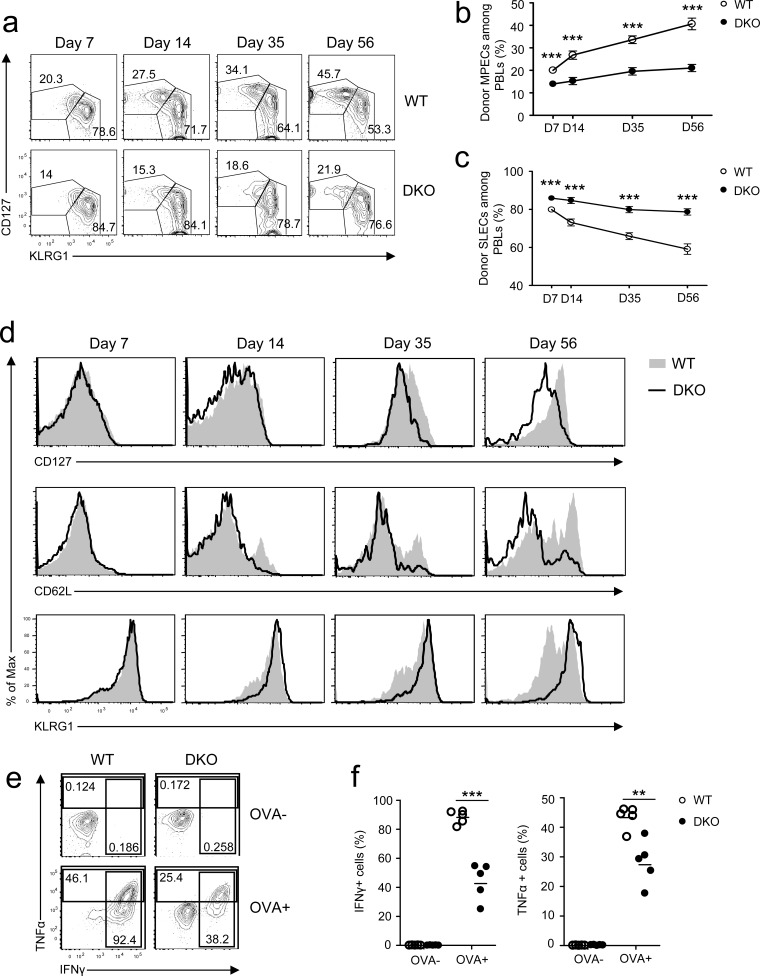
Effects of DGKαζ deficiency on CD8 effector/memory lineage differentiation and function Recipient mice injected with WT and DKO OT 1 T cells were similarly infected with *LM-OVA* and analyzed at the indicated times, as in Figure 1. **a.** Representative dot plots showing CD127 and KLRG1 expression in gated donor-derived OT1 T cells from PBLs. **b.**-**c.** Percentages of MPECs **b.** and SLECs **c.** of donor OT1 T cells at different times. **d.** Expression of cell surface markers in gated donor-derived OT1 T cells. **e.**-**f.** Splenocytes from recipient mice 7 days after *LM-OVA* infection were left unstimulated or stimulated with SIINFEKL peptide (1μg/ml) in the presence of GolgiPlug for 5 hours. IFNγ and TNFα were detected by intracellular staining. **e.** Repesentative dot plot of IFNγ and TNFα staining in gated OT1 T cells. **f.** Percentages of IFNγ- or TNFα-expressing OT1 cells. Data shown in **a.**-**d.** and **e.**-**f.** are representative of three and two independent experiments, respectively. **, *P* < 0.01; ***, *P* < 0.001 (Student's *t* test).

During a primary response, CD8 effector T cells differentiate into CD127^hi^KLRG1^lo^ MPECs and CD127^lo^KLRG1^hi^ SLECs [1, 2]. On day 7 after infection, MPEC and SLEC frequencies of donor-derived DKO OT1 T cells were slightly lower and higher than their WT counterparts, respectively (Figure 2a-2c). As time after infection increased, MPEC and SLEC frequencies of WT donor OT1 cells progressively increased and decreased, respectively. Such trends occurred in smaller magnitudes within DKO OT1 cells, however, leading to greater differences between WT and DKO OT1 T cells on day 56 after infection. Expression of CD127, CD62L, and KLRG1 was comparable between WT and DKO OT1 cells on day 7 at the peak of infection (Figure 2d); however, after day 14 of infection, CD127 expression was lower and KLRG1 expression was higher in DKO OT1 T cells than in WT OT1 T cells. DKO CD62L^+^ central memory (CM) T cells were also decreased compared with WT controls (Figure 2d). These observations indicated that DKO CD8 T cells had a reduced ability to form long-lived memory cells and instead were biased toward a short-lived effector program.

One important functional aspect of effector CD8 T cells is producing cytokines to control infection. To assess whether DGKα and ζ deficiency affected cytokine production by effector CD8 T cells during *LM-OVA* infection, splenocytes of OT1 T cell recipient mice 7 days after *LM-OVA* infection were stimulated with SIINFEKL peptide *in vitro* for 4-5 hours, followed by intracellular staining for TNFα and IFNγ. About 50% fewer DKO OT1 T cells expressed IFNγ and TNFα than WT OT1 T cells (Figure 2e-2f). Thus, DKO effector OT1 T cells were impaired in effector function.

### DGKα and ζ double deficiency impaired effector cell proliferation but not survival

**Figure 3:**
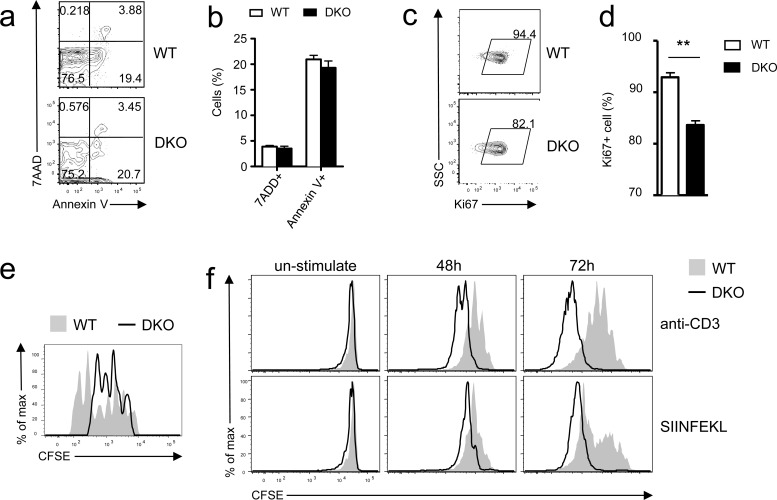
Differential effects of DGKαζ deficiency on OT1 T cell expansion *in vitro* and *in vivo* **a.**-**e.** Impaired expansion of DKO OT1 T cells following *LM-OVA* infection. **a.**-**b.** Frequencies of 7-AAD^+^ and Annexin V^+^ donor-derived OT1 T cells in PBLs on day 7 after *LM-OVA* infection. **c.**-**d.** Percentages of Ki67^+^ donor-derived OT1 T cells in PBLs on 6 day after infection (N = 7; **, *P* < 0.01 determined by Student's *t* test). **e.** *In vivo* assessment of donor-derived OT1 T cell proliferation. CFSE-labeled WT and DKO naïve OT1 T cells were adoptively transferred into recipient mice, followed by *LM-OVA* infection. Overlaid histograms show CFSE intensity in donor-derived OT1 T cells in splenocytes 72 hours after *LM-OVA* infection. Data shown are representative of two experiments. **f.** Enhanced TCR-induced DKO OT1 T cell proliferation *in vitro*. WT and DKO OT1 T cells from the spleen were CFSE labeled and stimulated with either anti-CD3 (0.1 μg/ml) or SIINFEKL peptide (1 ng/ml) *in vitro* for 48 and 72 hours. Overlaid histograms show CFSE intensity in gated WT and DKO OT1 T cells. Data are representative of two experiments.

Both defective proliferation and increased death may cause impaired *in vivo* responses of CD8 T cells. Donor DKO OT1 T cells did not show an obvious increase of death, reflected by similar frequencies of 7AAD^+^ and annexin V^+^ cells, compared with WT controls on day 7 after *LM-OVA* infection (Figure 3a-3b). By contrast, the frequencies of Ki67^+^ cells within adoptively transferred DKO donor OT1 T cells were lower than their WT counterparts (Figure 3c-3d), suggesting decreased expansion of DKO donor OT1 T cells. To further evaluate if DKO T cell proliferation was affected during the early phase of CD8 T cell response to *LM-OVA* infection, we transferred CFSE-labeled naïve WT and DKO OT1 T cells into recipient mice. DKO OT1 T cells proliferated much less than WT OT1 T cells 72 hours after *LM-OVA* infection (Figure 3e). Thus, impaired proliferation, but not survival, was responsible for failed accumulation of DKO CD8 T cells during primary response to bacterial infection *in vivo*.

The blunted *in vivo* response of DKO OT1 T cells was surprising because DGKα or ζ single-knockout T cells demonstrated enhanced responses *in vitro* and *in vivo* [26, 27]. Moreover, DKO OT1 T cells proliferated more vigorously than WT OT1 T cells *in vitro* after being stimulated with either anti-CD3 or SIINFEKL peptide for 48 or 72 hours (Figure 3f). Together, these results indicated that DGKαζ double deficiency had strikingly opposite effects on CD8 T cell expansion *in vivo* and *in vitro*. Although it caused defective expansion of these cells *in vivo* following microbial challenge, it enhanced T cell proliferation *in vitro* following TCR stimulation.

### Deficiency of DGKα and ζ impaired early priming of CD8 T cells

**Figure 4:**
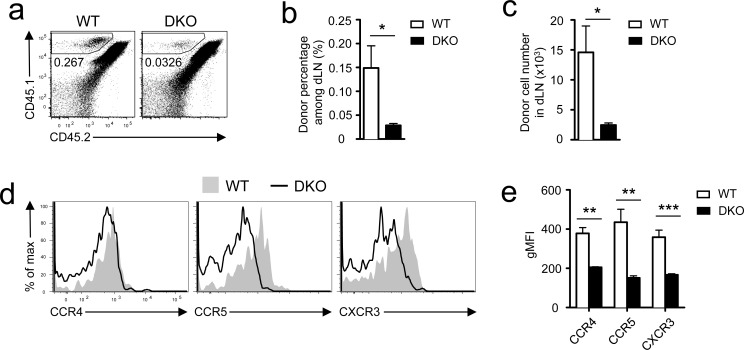
Assessment of DKO CD8 T cell priming *in vivo* CD45.1^+^ WT or DKO OT1 T cells were adoptively transferred into recipients (CD45.1^+^CD45.2^+^), which were subsequently immunized with SIINFEKL peptide emulsified in the CFA at inguina by hypodermic injection. Donor-derived OT1 T cells in inguinal dLNs harvested 12 hours after immunization were analyzed by flow cytometry. **a.** Representative dot plots; gated CD45.1^+^CD45.2^−^ cells represent donor-derived OT1 T cells. **b.** Mean ± SEM of percentages of donor-derived OT1 T cells (*n* = 5). **c.** Donor OT1 T cell numbers (mean ± SEM) in dLNs (*n* = 5). **d.**-**e.** Expression of indicated chemokine receptors in donor-derived OT1 T cells. **d.** Representative overlaid histograms. **e.** gMFI (mean ± SEM) of indicated chemokine receptors in gated WT or DKO OT1 T cells (*N* = 5). *, *P* < 0.05; **, *P* < 0.01; ***, *P* < 0.001 (Student's *t* test). Data shown are representative or calculated from two independent experiments.

The paradoxically different *in vitro* and *in vivo* responses of DKO OT1 T cells to antigen stimulation prompted us to examine whether DGKα and ζ regulate early priming of CD8 T cells. We injected 10 million CD45.1^+^ WT or DKO OT1 T cells into WT CD45.1^+^CD45.2^+^ recipients, which were subsequently immunized with SIINFEKL peptide emulsified in the complete Freund's adjuvant (CFA). Twelve hours after immunization, DKO OT1 T cell numbers in the dLNs were much lower than WT OT1 T cells (Figure 4a-4c). Chemokine receptors CCR4 and CCR5 play crucial roles in recruiting naïve CD8 T cells to CD4^+^ T cell/DC clusters and quantitatively and qualitatively promote CD8 T cell responses [32–35]. Both receptors were expressed at lower levels in DKO OT1 T cells than in WT controls (Figure 4d-4e). Furthermore, CXCR3, whose signal brings newly activated CD8 T cells to sites of ongoing infection [33], was also decreased in DKO OT1 T cells (Figure 4d-4e). Thus, DKO CD8 T cells were impaired in upregulating chemokine receptors, which might contribute to reduced migration to or retention in dLNs of these cells at the initial stage of bacterial infection and subsequently lead to blunted CD8 T cell response *in vivo*. Future studies should determine how DGKαζDKO reduced expression of these chemokine receptors in CD8 T cells.

### Critical role of DGKα and ζ for memory CD8 T cell homeostasis

**Figure 5:**
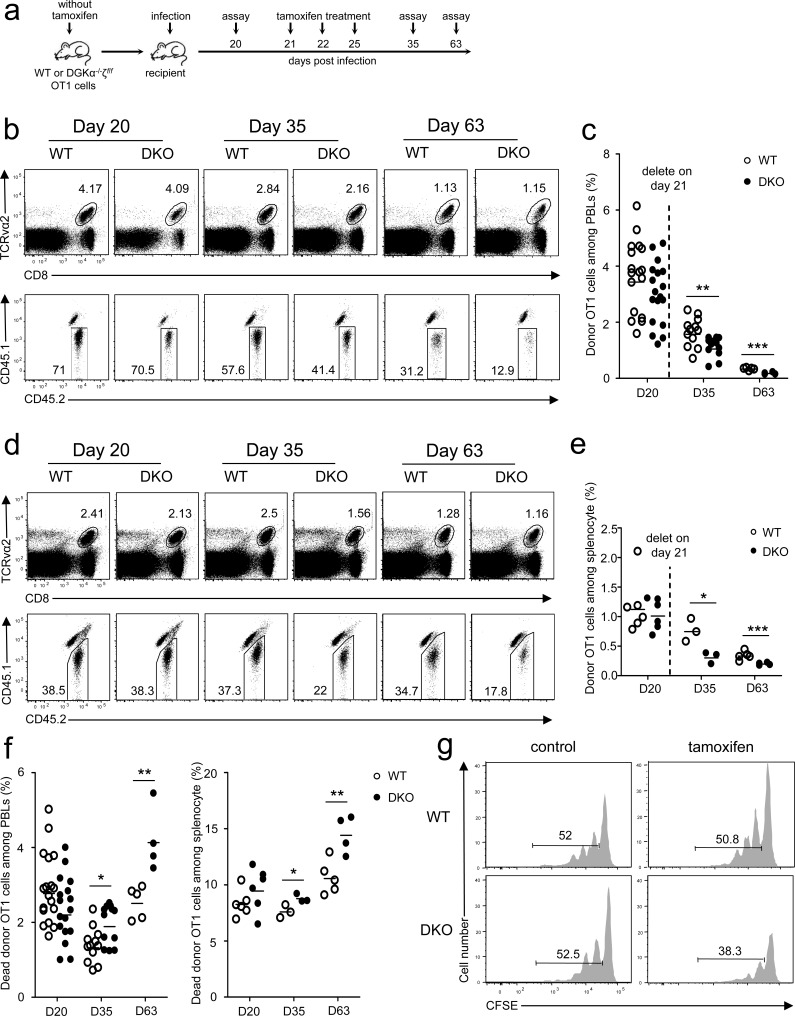
Critical role of DGKα and ζ in memory CD8 T cell homeostasis **a.** Experimental design for Figure 5b-5f. Sorted WT and DKO naïve OT1 T cells from mice without tamoxifen treatment were injected into CD45.1^+^CD45.2^+^ recipients. One day after injection, recipients were infected with *LM-OVA* (day 0); bled on day 20; injected with tamoxifen on days 21, 22, and 25; and examined on days 35 and 63. (b-d) Representative dot plots of WT and DKO donor OT1 cells in PBLs **b.** and splenocytes **d.** of recipients at indicated times postinfection. **c.**-**e.** Percentage of donor OT1 cells among PBLs **c.** and splenocytes **e.** at indicated times. Each circle represents one mouse. **f.** Ablation of DGKα and ζ in memory CD8 T cells increased their death. **g.** DGKαζDKO impaired memory CD8 T cell homeostatic proliferation. WT or DKO (without tamoxifen treatment) memory OT1 T cells isolated from splenocytes of primary recipients 30 days after *LM-OVA* infection were labeled with CFSE and adoptively transferred into secondary naïve recipients. These recipients were i.p. injected with tamoxifen on days 1, 2, and 5 after secondary transfer. Donor-derived OT1 memory cells in splenocytes were analyzed for CFSE dilution 30 days after secondary transfer. Data shown represent two independent experiments. *, *P* < 0.05; **, *P* < 0.01; ***, *P* < 0.001 (Student's *t* test).

Data in Figure 1f showed that the longer after infection, the bigger the differences between DKO and WT OT1 T cells. Moreover, more donor-derived DKO OT1 T cells were 7-AAD^+^ or annexin V^+^ than WT controls 35 days after infection (Figure S3a-S3b), suggesting that although DKO did not affect CD8 T cell survival during the early expansion phase (Figure 3a-3b), it increased death of memory CD8 T cells. Although these observations suggested that DGKαζDKO might affect memory CD8 T cell formation/maintenance, they did not directly address the impact of DKO on memory CD8 T cells. To this end, we adoptively transferred WT or DGKα^−/−^ζ*^f^*^/^*^f^*-ERCre naïve OT1 T cells from donor mice without tamoxifen treatment into recipient mice that were infected with *LM-OVA* on the following day (Figure 5a). Twenty days after infection, donor-derived WT and DKO OT1 T cells were similar in frequencies in both blood (Figure 5b-5c) and spleen in the recipients (Figure 5d-5e). Recipients were then i.p. injected with tamoxifen on days 21, 22, and 25 after infection (Figure 5a). On days 35 and 63, DKO OT1 T cells were about 30% to 60% lower than WT controls in PBLs (Figure 5b-5c) and splenocytes (Figure 5d-5e), indicating that DGKα and ζ are important for memory CD8 T cell maintenance.

Survival and homeostatic proliferation are important for long-term memory maintenance and self-renewal [36–39]. Although DKO memory OT1 T cells did not show an obvious survival defect on day 20 before tamoxifen injection, their death rates increased slightly at 2 weeks and obviously at 6 weeks after tamoxifen injection compared with WT controls (Figure 5f), suggesting that DGKαζ promote memory CD8 T cell survival. To determine if DGKαζ control memory CD8 T cell homeostatic proliferation, WT or DGKα^−/−^ζ*^f^*^/^*^f^*-ERCre OT1 T cells from donor mice without tamoxifen treatment were transferred into CD45.1^+^CD45.2^+^ hosts, followed by *LM-OVA* infection. Thirty days after infection, donor-derived WT or DGKα^−/−^ζ*^f^*^/^*^f^*-ERCre memory OT1 T cells were isolated, labeled with CFSE, and injected into secondary naïve recipients. Recipients were either treated with PBS or tamoxifen, and donor-derived memory OT1 T cells were analyzed 30 days later. Comparable donor WT and DGKα^−/−^ζ*^f^*^/^*^f^*-ERCre memory OT1 T cells (about 52%) underwent homeostatic proliferation in hosts treated with PBS (Figure 5g, left panels). However, fewer DKO OT1 memory T cells proliferated than WT OT1 memory T cells in tamoxifen-treated hosts (Figure 5g, right panels). Thus, ablation of both DGKα and ζ also impaired CD8 memory T cell proliferative self-renewal.

Together, deficiency of both DGK α and ζ impaired memory CD8 T cell homeostasis due to, at least, increased death and reduced homeostatic proliferation.

### Differential effects of DGKαζ double deficiency on memory CD8 T cell expansion and effector function in recall responses

**Figure 6:**
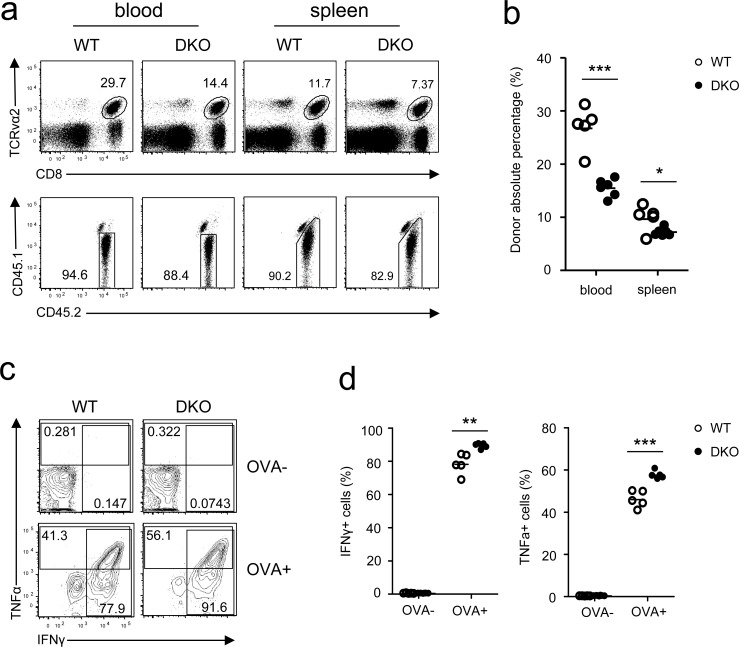
Effects of DGKα and ζ double deficiency on memory CD8 T cell responses WT and DKO memory OT1 T cells sorted from splenocytes of recipients on day 35 after *LM-OVA* infection were transferred into secondary recipients (1 × 10^4^/mouse), which were subsequently infected by *LM-OVA* 18 hours after transfer. **a.** Representative dot plots and percentages **b.** of WT and DKO donor-derived CD45.1^−^CD45.2^+^ memory OT1 cells in PBLs and splenocytes in secondary recipients 7 days postinfection. **c.**-**d.** Splenocytes from secondary recipients were stimulated with SIINFEKL peptide in the presence of GolgiPlug for 5 hours, followed by cell surface and intracellular staining and FACS analysis. Representative contour plots **c.** and percentages of IFNγ^+^ or TNFα^+^ cells **d.** of donor-derived WT and DKO memory OT1 T cells are shown. Data shown represent two independent experiments. *, *P* < 0.05; **, *P* < 0.01; ***, *P* < 0.001 (Student's *t* test).

To examine how DGKα and ζ double deficiency affected memory CD8 T cell responses, we sorted WT and DKO OT1 donor-derived memory cells from recipient mice 5 weeks after *LM-OVA* infection and adoptively transferred these cells into naive CD45.1^+^CD45.2^+^ recipients, followed by *LM-OVA* infection. Similar to primary responses, DKO memory CD8 T cell frequencies in both PBLs and splenocytes in recipients were lower than their WT counterparts 7 days after infection (Figure 6a-6b). Unlike primary responses, however, TNFα and IFNγ production by *LM-OVA* rechallenged DKO OT1 memory T cells was slightly increased compared to WT controls following *in vitro* SIINFEKL stimulation (Figure 6c-6d). Thus, DGKα and ζ also promoted memory CD8 T cell expansion after reencountering the same bacterial pathogen but weakly inhibited their effector function.

### Contribution of decreased miR-155 expression to compromised responses of DGKαζDKO CD8 T cells

**Figure 7:**
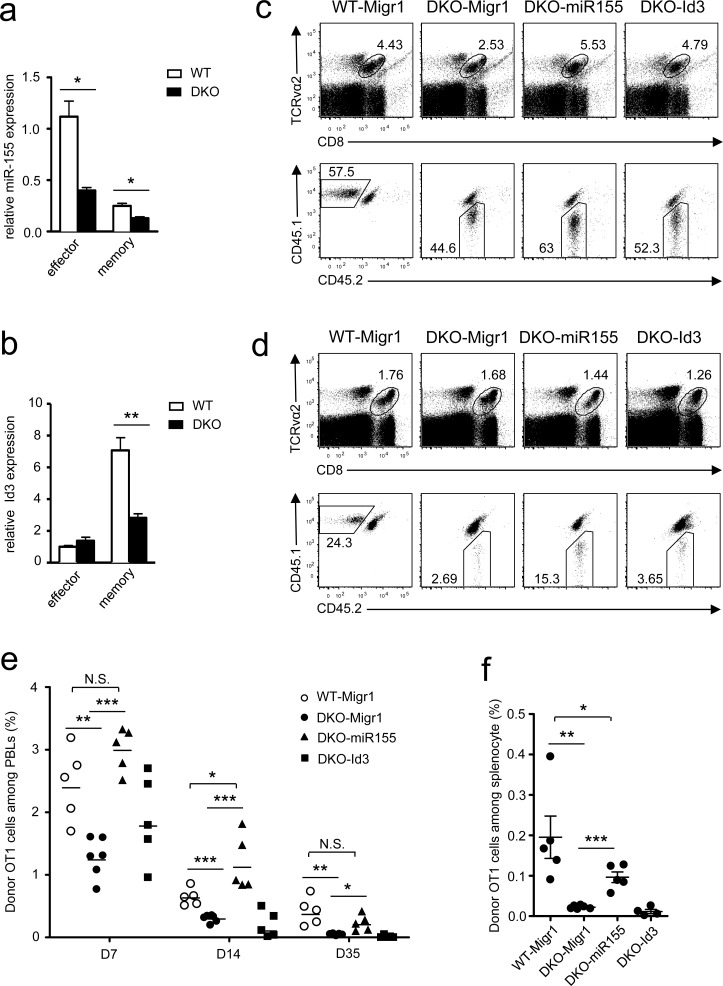
Overexpression of miR-155 restored DKO CD8 T cell responses **a.**-**b.** Expression of miR-155 **a.** and Id3 **b.** in donor-derived OT1 effector or memory cells sorted from splenocytes of recipients on day 7 or 35 after *LM-OVA* infection. Data shown (mean ± SEM) are calculated from triplicate and represent three experiments. **c.**-**d.** Splenocytes from WT or DKO OT1 mice were spin-infected with indicated retrovirus after SIINFEKL stimulation for 40 hours. Infected GFP^+^ OT1 T cells were adoptively transferred into recipients, followed by infection with *LM-OVA*. Representative dot plots of WT or DKO donor-derived OT1 cells in PBLs of recipients on day 7 **c.** and day 35 **d.** postinfection are shown. **e.** Percentage of donor-derived OT1 cells among PBLs at indicated times postinfection. **f.** Percentage of donor OT1 cells in splenocytes on day 35 postinfection. *, *P* < 0.05; **, *P* < 0.01; ***, *P* < 0.001 (Student's *t* test). Data in **c.**-**f.** represent two experiments.

CD8 T cell-mediated responses are tightly controlled by multiple transcription factors, metabolic programming, and microRNAs [17, 40–45]. A panel of key transcription factors and enzymes in DKO OT1 T cells on day 7 after *LM-OVA* infection were expressed at levels similar to WT controls, with the exception of elevated Eomes and hexokinase 2 (HK2, Figure S4) but decreased miR-155 and Id3 in DKO OT1 T cells. miR-155 obviously decreased in both effector and memory DKO OT1 T cells (Figure 7a), but Id3 decreased only in DKO memory CD8 T cells (Figure 7b).

Because both miR-155 and Id3 participate in programming and regulating CD8 T cell responses [21, 41, 46, 47], we investigated whether decreased expression of these molecules contributed to defective DKO CD8 T cell responses *in vivo*. To this end, we transduced DKO OT1 T cells with retrovirus-expressing miR-155 plus GFP, Id3 plus GFP, or GFP alone following stimulation with SIINFEKL peptide. GFP^+^-transduced OT1 cells were adoptively transferred into recipient mice, followed by *LM-OVA* infection. The frequencies of miR-155-expressing DKO OT1 T cells in PBLs were obviously higher than those expressing GFP alone on day 7 (Figure 7c, 7e), day 14 (Figure 7e), and day 35 (Figure 7d-7e) after infection and were restored to levels similar to WT OT1 T cells. Similar results were also observed in the spleen on day 35 after *LM-OVA* infection (Figure 7f). In contrast to miR-155, overexpression of Id3 failed to restore DKO OT1 T cell expansion and memory T cell formation (Figure 7c-7f). Taken together, these results suggest that decreased miR-155 expression in DKO OT1 T cells contribute to defective effector CD8 T cell expansion and memory CD8 T cell formation and maintenance.

### Elevated SOCS1 and impaired γ-chain cytokine receptor signaling in DKO CD8 T cells

**Figure 8:**
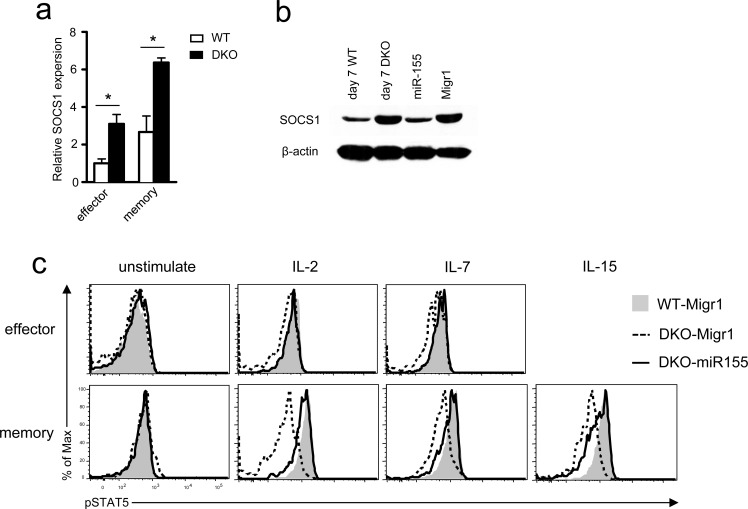
Impaired γ-chain cytokine signaling in DKO CD8 T cells **a.** SOCS1 mRNA levels in donor-derived effector and memory OT1 T cells sorted from splenocytes of recipients on day 7 (effector) or 35 (memory) after *LM-OVA* infection determined by real-time qPCR. **b.** SOCS1 protein levels detected by Western blot analysis. Left two lanes: lysates from WT or DKO donor-derived OT1 cells sorted from splenocytes of recipients on day 7 after *LM-OVA* infection; right two lanes: lysates from *in vitro* cultured WT OT1 cells infected with either miR-155 or control retrovirus (Migr1). **c.** STAT5 phosphorylation in effector and memory OT1 T cells. WT and DKO OT1 T cells transduced with retrovirus expressing GFP alone (Migr1) or GFP plus miR-155 were adoptively transferred into recipient mice, followed by *LM-OVA* infection. Seven and 35 days after infection, splenocytes were stimulated with IL-2 (100U/mL), IL-7 (10ng/mL), or IL-15 (10ng/mL) for 18 minutes, and STAT5 phosphorylation in donor-derived OT1 cells was examined by intracellular staining and FACS analysis. Overlaid histograms show STAT5 phosphorylation in effector (day 7) and memory (day 35) OT1 T cells. *, *P* < 0.05; **, *P* < 0.01; ***, *P* < 0.001 (Student's *t* test). Data shown represent two experiments.

miR-155 targets SOCS1 to enhance γ-chain cytokine signaling including STAT5 phosphorylation [21]. Consistent with decreased miR-155 expression, SOCS1 mRNA increased in DKO effector and memory OT1 T cells compared with WT controls (Figure 8a). The same was true for SOCS1 protein in DKO OT1 T cells isolated from recipients 7 days after *LM-OVA* infection (Figure 8b). Concordantly, STAT5 phosphorylation in DKO effector and memory OT1 T cells was lower than in WT controls following IL-2, IL-7, or IL-15 stimulation (Figure 8c). Importantly, miR-155 overexpression in OT1 T cells reduced SOCS1 expression (Figure 8b) but increased STAT5 phosphorylation in DKO OT1 T cells to levels similar to WT controls (Figure 8c), indicating that decreased miR-155 expression led to elevated SOCS1 protein levels and impaired γ-chain cytokine receptor signaling in DKO OT1 T cells.

### Absence of DGKα and ζ impaired TCR-induced NFκB-mediated miR-155 expression

**Figure 9:**
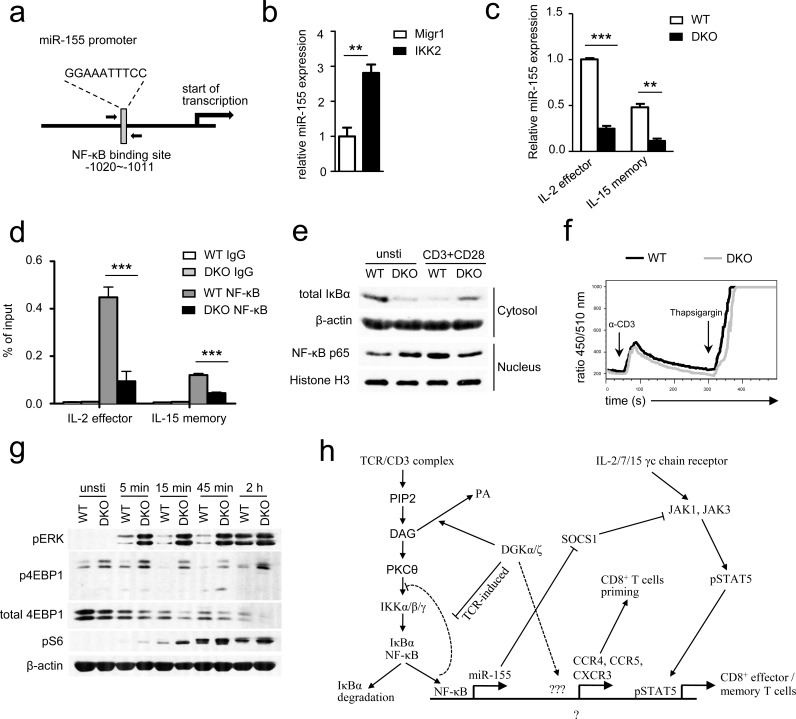
DGKα and ζ regulate miR-155 transcription, SOCS1 expression, and γ-chain cytokine signaling by modulating TCR-induced NFκB activation **a.** NFκB binding site in the miR-155 promoter identified by TFSEARCH ver.1.3. **b.** Enhanced IKKβ activity increased miR-155 expression in OT1 T cells. Splenocytes from WT OT1 mice were transduced with CA-IKKβ retrovirus. Total RNA from sorted GFP^+^ transduced cells were isolated and used for RT-qPCR. Bar graphs are mean ± SEM of triplicates and represent 2 experiments. **c.** Expression of miR-155 in cultured IL-2-induced effector and IL-15-induced memory OT1 T cells. Splenocytes from WT or DKO OT1 mice were activated with SIINFEKL (10ng/ml) and IL-2 (100 U/ml) for 3 days and subsequently cultured in the presence of either IL-2 or IL-15 (10 ng/ml) alone for another 4 days to generate effector-like or memory-like CD8 T cells, respectively. Viable cells were sorted for real-time qPCR analysis. **d.** *In vitro* cultured effector and memory WT or DKO OT1 T cells were subjected to ChIP with an anti-NFκB antibody or control IgG, followed by qPCR using primers flanking the NFκB binding site in the miR-155 promoter. Data are presented as mean ± SEM of percentages of immunoprecipitated DNA relative to the total input DNA of the miR-155 promoter and are representative of two independent experiments. **e.** Immunoblotting analysis of nuclear and cytoplasmic fractions of WT and DKO T cells for NFκB activation. OT1 T cells purified from splenocytes of WT or DKO OT1 mice were left unstimulated or were stimulated for 2 hours with anti-CD3 and anti-CD28 antibodies. Nuclear and cytoplasmic fractions were isolated and subjected to immunoblotting analysis. **f.** Ca^++^ influx WT and DKO splenocytes OT1 T cells determined by flow cytometry based on the change in the FL5/FL4 ratio after cross-linking of TCRs, CD4 and CD8 on Indo-1-loaded T cells. **g.** immuno-blotting of WT and DKO CD8^+^ T cells stimulated with or without plate-bound anti-CD3 and anti-CD28 for different times with indicated antibodies. Data shown are representative of two experiments. *, *P* < 0.05; **, *P* < 0.01; ***, *P* < 0.001 (Student's *t* test). **h.** DGK controls DAG-mediated signaling downstream of the TCR and bridge TCR signaling and γ-chain cytokine receptor signaling to regulate CD8 effector and memory T cell differentiation. Please see text in Discussion for details.

We next sought to determine the mechanism by which DGKα and ζ control miR-155 expression in effector/memory CD8 T cells. NFκB has been reported to activate miR-155 transcription in B cells, Burkitt lymphoma, and acute myeloid leukemia [48–51]. However, its role for miR-155 expression in T cells has not been reported. In the miR-155 promoter, a consensus NFκB binding is located at −1020 to −1011 bp upstream of its transcription starting site (Figure 9a). Expression a constitutively active IKKβ in OT1 T cells increased miR-155 expression (Figure 9b), suggesting involvement of NFκB in activating miR-155 transcription in these cells. To determine whether NFκB indeed bound to this site in T cells, we differentiated WT and DKO effector and memory OT1 T cells using IL-2 and IL-15, respectively, according to a published protocol [52]. Consistent with *in vivo* data shown in Figure 7a, DKO effector and memory OT1 T cells generated in this *in vitro* system also had decreased miR-155 expression (Figure 9c). Chromatin immunoprecipitation (ChIP) showed NFκB association to the miR-155 promoter in WT OT1 T cells (Figure 9d). However, this association was substantially decreased in DKO effector and memory OT1 T cells, suggesting impaired NFκB activation in these cells. These results are surprising because it have been demonstrated that DAG associates with and activates PKCθ, which leads to activation of the IKK complex, subsequent IκBα phosphorylation and degradation, and NFκB nuclear translocation, and that DGK activity inhibits DAG-mediated signaling [9–11, 53]. In DGKα^−/−^ or DGKζ^−/−^ single knockout T cells, TCR induced IκBα and NFκB phosphorylation was elevated (Figure S5), suggesting enhanced activation of the PKCθ-IKK-NFκB pathway in these cells. To assess this pathway in DKO OT1 T cells, we isolated cytosolic and nuclear fractions in WT and DKO OT1 T cells before and after TCR + CD28 stimulation for 2 hours. Without TCR stimulation, DKO OT1 T cells indeed displayed decreased IκBα protein and increased NFκB nuclear localization (Figure 9e), suggesting elevated activation of the PKCθ-IKK-NFκB pathway. However, when stimulated by anti-CD3 and anti-CD28, DKO OT1 T cells contained higher levels of IκBα in the cytosol but less nuclear NFκB than WT controls, suggesting impaired activation of the PKCθ-IKK-NFκB pathway. Thus, although DKO OT1 T cells contained a low-grade constitutive NFκB activation, they were defective in TCR/CD28-induced NFκB activation, leading to decreased association to the miR-155 promoter for its transcription.

We further examined TCR induced calcium (Ca^++^) influx in DKO OT1 T cells to determine if proximal TCR signaling events were impaired in these cells. As shown in Figure 9f, TCR induced Ca^++^ influx in DKO OT1 T cells was not obviously different from WT controls (Figure 9f), suggesting that PLCγ activation and subsequent inositol trisphosphate production were not impaired in DKO OT1 T cells. As mentioned earlier, DAG also activates the RasGRP1-Ras-Erk1/2 pathway. In contrast to NFκB activation, TCR induced Erk1/2 phosphorylation was elevated in DKO OT1 T cells (Figure 9g). Concordantly, TCR-induced S6 and 4EBP-1 phosphorylation, an mTOR complex 1 dependent event known downstream of the DAG-RasGRP1-Ras-Erk1/2 pathway in thymocytes [12], was also enhanced in DKO OT1 T cells. Interestingly, TCR engagement induced 4EBP-1 degradation in WT OT1 T cells that was most obvious 2 hours after stimulation. Such 4EBP-1 degradation was accelerated in DKO OT1 T cells. Because 4EBP-1 sequesters eIF4E from cap-dependent translation initiation and 4EBP-1 phosphorylation releases eIF4E, the down regulation of 4EBP-1 after TCR engagement might provide an additional mechanism to promote protein translation during T cell activation. Thus, DGKα and ζ double deficiency differentially affected TCR-induced activation of two important signal cascades downstream of DAG; it impaired activation of the PKCθ-IKK-NFκB pathway but enhanced activation of the RasGRP1-Ras-Erk1/2-mTOR pathway.

## DISCUSSION

Understanding how CD8 T cells differentiate into effector and memory cells is important for improving treatment of infectious diseases and tumors and for enhancing vaccination efficacy. Although previous studies with DGKα or ζ single-deficient mice have found that DGK activity inhibits T cell activation and proliferation as well as primary CD8 T cell-mediated immune response *in vivo*, our current study surprisingly reveal that simultaneous ablation of both DGKα and ζ severely impair CD8 T cell-mediated responses to bacterial infection, manifested by defective expansion of effector and formation of memory CD8 T cells after bacterial challenge and by impaired maintenance of CD8 memory T cells. Our data indicate the presence of at least 2 DGK isoforms in CD8 T cells as an evolutionary advantage so that loss-of-function mutations in one DGK isoform do not compromise CD8 T cell functions and host immunity against pathogens.

Our data suggest that ablation of both DGKα and ζ affects CD8 T cell responses at multiple stages. First, it caused defective early priming of CD8 T cells. At the earliest hours after infection, DGK activity ensured recruitment of naïve CD8 T cells to the dLNs *via* upregulating CCR4, CCR5, and CXCR3. Second, it compromised CD8 T cell proliferation *in vivo*. Because DKO CD8 T cells proliferated more vigorously than WT controls *in vitro* following TCR engagement, DKO CD8 T cells might not have an intrinsic defect in proliferative potential. We speculate that impaired CD8 proliferation *in vivo* may have resulted from reduced engagement of CD8 T cells with antigen-presenting cells. Third, ablation caused severe reduction of CD8 memory T cells after *LM-OVA* infection. CD8 T cell deficiency of either DGKα or ζ displayed increased potential to develop to SLECs but decreased potential to develop to MEPCs during LCMV infection [28]. DKO CD8 T cells also showed similar trends during primary responses to *LM-OVA* challenge. Besides severely reduced expansion of DKO CD8 T cells, decreased MEPC differentiation may also contribute to their decreased memory formation. Transcription factor T-bet promotes SLEC differentiation, while Eomes enhances MEPC formation and memory responses [7, 54, 55]. However, T-bet expression is not obviously different between WT and DKO CD8 T cells, whereas Eomes increases in DKO CD8 T cells, suggesting that DKO may not affect CD8 SLEC/MEPC differentiation *via* these transcription factors. Memory CD8 T cells have the ability to self-renew by homeostatic proliferation, which is essential for long-lived memory cell maintenance [40, 45]. We have found that ablation of both DGKα and ζ in preformed CD8 memory T cells caused increased death but impaired homeostatic proliferation, which may also contribute to reduction of memory CD8 T cells.

DGKα or ζ single-knockout mice display enhanced CD8 cell function, particularly during primary challenges. Absence of DGKα and ζ has also been shown to enhance chimeric antigen receptor (CAR)-transduced T cell-mediated antitumor immunity in mice [28, 56, 57]. Based on these observations, it has been proposed that inhibition of DGK activity can boost T cell activation and enhance immunity against microbial pathogens and tumors. Although how DGKα and ζ double deficiency differentially affects CAR-T cell mediated anti-tumor immunity and CD8 T cell mediated anti-microbial immune responses is unclear at present. Our data presented in this study raise concerns about potentially undesirable effects of DGK inhibition. Indiscriminate inhibition of multiple DGK isoforms might lead to detrimental outcomes, such as compromised CD8 T cell responses (this study), T cell developmental blockade in the thymus [58], and multi-organ autoimmune diseases (manuscript in preparation). Isoform-specific DGK inhibitors should be advantageous for modulating immune responses for therapeutic purposes, which are unlikely to have severe consequences.

DAG-mediated activation of the RasGRP1-Ras-Erk1/2 and PKCθ-IKK-NFκB pathways plays an important role in T cell activation. Previous studies *via* overexpression or genetic ablation of either DGKα or ζ have demonstrated that these DGKs negatively control DAG-mediated signaling in T cells [10, 23, 24, 27]. Consistent with this notion, we have found that DKO CD8 T cells display a low-grade constitutive NFκB activation. Unexpectedly, these cells are impaired in TCR- and CD28-induced NFκB activation. Because expression of a constitutive IKKβ also causes abnormalities in CD8 T cell responses similar to DKO, which include defective NFκB activation and impaired effector T cell expansion and memory formation following *LM-OVA* infection [59], we speculate that DKO T cells may trigger a negative feedback mechanism due to dysregulated DAG-PKC/IKK activity. Such negative feedback mechanisms are likely downstream of DAG and selectively to the PKCθ-IKK-NFκB pathway as TCR induced calcium influx is not impaired and TCR induced Erk1/2 activation is actually enhanced in DKO T cells. Additionally, triggering such negative feedback mechanisms likely requires ablation of both DGKα and ζ as miR-155 expression was not decreased in DGKα or ζ deficient OT1 T cells (Figure S6). Future studies should illustrate the exact mechanisms by which DKO causes such paradoxical inhibition of TCR-induced NFκB activation.

Both expansion of CD8 effector T cells and formation/maintenance of memory CD8 T cells are influenced by the cytokine milieu [60, 61]. IL-15, IL-7, and IL-2 signal through receptors sharing a common γ-chain to control CD8 effector and memory responses [20, 38, 39, 62, 63]. IL-2 and IL-15 are critical for the differentiation of effector and memory CD8 T cells, respectively [19, 20]. IL-7 is important for memory CD8 T cell homeostasis and longevity [37, 52, 64]. SOCS1 negatively controls signaling from these γ-chain cytokine receptors [65]. Previous studies have demonstrated the crucial role of miR-155 in regulating CD8 T cell responses by targeting SOCS1 to ensure γ-chain cytokine signaling [21, 46]. Deficiency of miR-155 causes defective expansion of effector CD8 T cells and generation of memory CD8 T cells due to increased SOCS1 expression [46]. We have shown that DKO caused defective CD8 T cell responses that correlate with decreased miR-155 expression and impaired γ-chain cytokine signaling. Moreover, we have provided the first evidence that NFκB directly binds to the miR-155 promoter in effector and memory CD8 T cells and that this association is diminished when DGKα and ζ are ablated. Importantly, defective effector expansion and memory formation of DGKαζDKO CD8 T cells, as well as impaired γ-chain cytokine signaling (STAT5 phosphorylation), can be rescued by overexpression of miR-155. Our data illustrate that DGKα and ζ control both effector CD8 T cell expansion and memory CD8 T cell generation and maintenance, mainly through promoting miR-155 expression to ensure γ-chain cytokine signaling.

On the basis of our current study and other previous reports [9, 21, 33], we propose that DGKαζ serve as crucial modulators to not only ensure proper NFκB activation but also bridge TCR signal to γ-chain cytokine signal in CD8 T cells. When both DGKα and ζ are ablated, elevated DAG due to tonic TCR signal or signals from other receptors may cause a low-grade PKCθ-IKK-NFκB activation, which may trigger negative feedback mechanisms that prevent full activation of this pathway after TCR engagement. Failure of NFκB nuclear translocation may cause decreased NFκB association to the miR-155 promoter and reduced miR-155 transcription, which may lead to elevated expression of one of its targets, SOCS1. Elevated SOCS1 expression may inhibit γ-chain receptor signaling including STAT5 phosphorylation. Additionally, decreased NFκB nuclear translocation may also contribute to decreased expression of other molecules involved in T cell activation and migration. These abnormalities may result in overall impaired DKO CD8 T cell expansion and memory formation/maintenance (Figure 9h).

## MATERIALS AND METHODS

### Mice

C57BL/6 mice, cogenic CD45.1^+^ mice, and Thy1.1^+^ mice were purchased from the Jackson Laboratory. DGKα^−/−^, DGKζ^−/−^, and ERCre mice were previously reported [26, 66). DGKζ*^f^*^/^*^f^* mice were generated by introducing two LoxP sites that were inserted between exons 9 and 10 and between exons 14 and 15 of the DGKζ locus (manuscript in preparation). TCR transgenic OT1 mice were purchased from the Jackson Laboratory and crossed with DGKα^−/−^ζ*^f^*^/^*^f^*-ERCre mice to generate DGKα^−/−^ζ*^f^*^/^*^f^*-OT1-ERCre mice in specific pathogen-free facilities at Duke University Medical Center. Experiments in this study were performed according to protocols approved by the Institutional Animal Care and Usage Committee of Duke University. WT OT1 mice and DGKα^−/−^ζ*^f^*^/^*^f^*-OT1-ERCre mice or WT OT1 and DGKα^−/−^ζ*^f/f^*-ERCre OT1 T cell recipient mice were i.p. injected with tamoxifen (100 mg/kg body weight) on days 1, 2, and 5 or on indicated days to delete DGKζ, and mice were then euthanized for experiments on day 8 or other indicated days.

### Reagents, plasmids, and antibodies

Iscove's modified Dulbecco's medium (IMDM) was supplemented with 10% (vol/vol) FBS, penicillin/streptomycin, and 50 μM 2-mercaptoethanol (IMDM-10). Fluorescence-conjugated anti-mouse CD8α [53-6.7), TCRVα2 (B20.1), CD25 (PC61), CD69 (H1.2F3), CD122 (TM-β1), CD127 (SB/199), CD44 (IM7), CD62L (MEL-14), Thy1.1 (OX-7), Thy1.2 (58-2.1), CD45.1 (A20), CD45.2 (104), CCR4 (2G12), CCR5 (HM-CCR5), CXCR3 (CXCR3-173), T-bet (4B10), IFN-γ (XMG1.2), TNFα (MP6-XT22), and Annexin V antibodies were purchased from BioLegend; anti-mouse Ki67, KLRG1 (2F1), and pSTAT5 (pY694) antibodies were purchased from BD Biosciences; anti-mouse Eomes (Dan11mag) antibodies were purchased from eBioscience. Cell death was determined by staining cells with 7-AAD or Live/Dead Fixable Violet Dead Cell Stain (Invitrogen). The coding region of Id3 cDNA was amplified from mouse Id3 cDNA (pBS/KS-mId3sp, Addgene) using a forward primer, 5′-CGGGATCCATGAAGGCGCTGAGCCCGGT-3′, and a reverse primer, 5′-CGGAATTCCACGACCGGGTCAGTGGCAA-3′. After digestion with Bam HI and EcoRI, the PCR product was cloned into BamH1 and EcoRI sites of the MIGR1-FLAG Vector [67]. CA-IKKβ (S177E and S181E) was released from an IKK-2 S177E S181E plasmid (Addgene) and cloned into Not I site of modified MIGR1 retroviral vector.

### Flow cytometry

Standard protocols were used to prepare single-cell suspensions from the blood, spleen, and lymph nodes of mice, and preparation of single-cell suspensions from born marrow, lung and liver was according to previous reports [68–70]. After lysis of red blood cells with the ACK buffer, cells were resuspended in IMDM-10 and subsequently stained with antibodies in PBS containing 2% FBS. Intracellular staining for Ki67 (BD Biosciences) was performed using the eBioscience Foxp3 Staining Buffer Set. Ki67 was detected with Alexa Fluor 488-conjugated goat anti-mouse IgG (H + L) (Invitrogen). Intracellular staining for IFNγ and TNFα was performed using the BD Biosciences Cytofix/Cytoperm and Perm/Wash solutions. STAT5 phosphorylation was detected according to a previous report [21]. Briefly, cells were stimulated with indicated cytokines for 18 minutes and fixed with 0.5% formaldehyde in PBS for 15 minutes. After washing with IMDM-10, cells were permeabilized in 80% methanol for 20 minutes, washed, and stained with an anti-phosphor-STAT5 antibody (BD Biosciences). Stained cells were collected on a BD FACSCanto II flow cytometer and analyzed using the Flowjo software.

### Purification of OT1 cells

Enrichment of TCRVα2^+^ cells from spleen and lymph node samples was performed using Miltenyi Biotec LS columns. Cells were incubated with PE-TCRVα2 antibody and then with anti-PE magnetic beads to isolate TCRVα2^+^ cells according to the manufacturer's protocol. Enriched samples were stained with anti-CD8, CD44, and CD62L antibodies and sorted on a MoFlo Astrios or FACS DiVa sorter to obtain viable OT1 naïve, effector, or memory cells. Congenical markers (CD45.1 and CD45.2 or Thy1.1 and Thy1.2) were used to identify donor-derived OT1 cells.

### Adoptive transfer and *LM-OVA* infection

A total of 1 × 10^4^ naïve, memory, or retroviral-infected OT1 cells in 200 μl serum-free IMDM were adoptively transferred by *i.v.* injection into sex-matched recipients. After 18-24 hours, recipient mice were injected *i.v.* with 100 μl 10^5^ CFU/mL *LM-OVA* [71]. For competitive adoptive transfers, 5 × 10^3^ WT cells were mixed with an equal number of DGKα^−/−^ζ*^f^*^/^*^f^*-ERCre OT1 cells before injection.

### IEL and LP preparation

IEL and LP were prepared as previously reported protocol with modification [72]. Briefly, after washed with PBS and removal of fat tissues and Payer's Patch (PP), the intestines were cut longitudinally followed by cut laterally into 0.5cm pieces. These pieces from the small intestine and colon were put into 20ml pre-warmed IEL preparation buffer (PBS with 10% FBS, 5mM EDTA and 1mM DTT) in a 50 ml tube or into a 15ml tube with 5ml IEL preparation buffer respectively and incubated at 37°C with constant orbital shaking (200rpm) for 30 min. After vortexed vigorously for 15 seconds, the pieces were filtered through a cell strainer and the pieces were treated the same way for a second time. Cells filtered through the strainer were combined and washed with PBS, and resuspended in complete IMDM medium as IELs. The pieces after IEL isolation were further cut into small pieces, and digested in a 50ml tube with 10ml pre-warmed digestion buffer (IMDM with 10% FBS, 1.5mg/ml collagenase IV and 0.5mg/ml DNase I) for small intestine or in a 15ml tube with 5ml digestion buffer for colon at 37°C, 200rpm for 30 min. Digested samples were filtered through cell strainers to collect LP cells. LP preparations were centrifuged and washed with IMDM twice and re-suspended in complete IMDM medium for FACS analysis.

### Real-time RT-PCR

Sorted viable cells were immediately lysed in Trizol for RNA preparation. cDNA was made using the iScript Select cDNA Synthesis Kit (Bio-rad). Real-time quantitative PCR was conducted and analyzed. mRNA levels of genes of interest were normalized with β-actin and calculated using the 2^−ΔΔCT^ method [73]. Primers used in this study are listed in Table 1.

**Table 1:** Real-time qPCR primer used in this study

Gene name	Forward primer (5′-3′)	Reverse primer (5′-3′)
T-bet	GGTGTCTGGGAAGCTGAGAG	GAAGGACAGGAATGGGAACA
Eomes	CCCTATGGCTCAAATTCCAC	TGGGGTTGAGTCCGTTTATG
Prdm1	TGGTATTGTCGGGACTTTGC	TGGGGACACTCTTTGGGTAG
STAT3	CAGTTCCTGGCACCTTGG	CAATTTCACCCAAGAGATTATGAA
miR-155	GCTGAAGGCTGTATGCTGTT	GGCCTTGTGTCCTGTTAATG
Id2	GCTTATGTCGAATGATAGCAAA	CTCCTGGTGAAATGGCTGAT
Id3	CACTTACCCTGAACTCAACGCC	CCCATTCTCGGAAAAGCCAG
Bcl-6	CTGCAGATGGAGCATGTTGT	CGGCTGTTCAGGAACTCTTC
IDH3a	AGGTTTTGCTGGTGGTGTTC	CCTGGTCCTTGAATTGCTGT
LDHb	GGTGAATGTGGCAGGAGTCT	CCCAGTTGGTGTAGCCTTTG
HK2	TGGGTTTCACCTTCTCGTTC	ACCACATCTCTGCCTTCCAC
Arg2	ATATGGTCCAGCTGCCATTC	AGGGATCATCTTGTGGGACA
GFPT1	TGTTCCTCGAACAAGACGAG	GTCATTGCCTCCGTCAAGTC
G6PDX	GCCTTCCACCAAGCTGATAC	GCATAGCCCACAATGAAGGT
PDHX	GAGCAAGTTGGAGGTGGTTT	CAATGTTCCCTTGCTCCATC
GOT2	AGAGGGCTCTTCCCACAACT	ACCGAGAACTCCTTGGTCAG
SOCS1	GAAGCCATCTTCACGCTG	ACACTCACTTCCGCACCTTC
β-actin	TGTCCACCTTCCAGCAGATGT	AGCTCAGTAACAGTCCGCCTAGA

### *In vitro* stimulation and cytokine assay

Three million splenocytes were incubated with 1 μg/ml of SIINFEKL (OVA_257-264_) peptide in the presence of 1 ng/ml of GolgiPlug for 4-5 hours, and frequencies of IFNγ and TNFα were analyzed by flow cytometry after staining with fluorochrome-conjugated antibodies.

For STAT5 phosphorylation, splenocytes were stimulated with IL-2 (100 U/mL), IL-7 (10 ng/mL), and IL-15 (10 ng/mL) at 37°C for 18 minutes before intracellular pSTAT5 staining.

### Proliferation and apoptosis assay

Naïve OT1 cells from WT or DGKα^−/−^ζ*^f^*^/^*^f^* (CD45.2^+^) OT1 donors were labeled with 10 μM CFSE at room temperature for 9 minutes. 1 × 10^5^ labeled WT or DKO OT1 cells were adoptively transferred into recipient mice (CD45.1^+^CD45.2^+^) and followed by *LM-OVA* infection on the following day. Seventy-two hours after infection, splenocytes were stained with surface markers before being analyzed by flow cytometry. Ten thousand naïve OT1 cells from WT or DGKα^−/−^ζ*^f^*^/^*^f^* OT1 donors were adoptively transferred as described above, and Ki67 staining was performed 6 days after *LM-OVA* infection.

For survival and apoptosis, after cell surface staining, splenocytes were stained for AnnexinV at room temperature for 15 minutes, and 7-AAD (Invitrogen) was added to the samples shortly before collection.

### Retroviral transduction

Retroviruses for Migr1, miR-155, Id3 or CA-IKKβ were made using the Phoenix-Eco packaging cell line. Three million OT1 cells in 24-well plates in 1 ml IMDM-10 were stimulated with SIINFEKL (100 ng/mL) and anti-CD28 (500 ng/mL) for 40 hours. After the 500 μl cultural medium with retroviral supernatants containing polybrene (5 μg/mL final concentration) were replaced, cells were spin-infected at 22°C, 1,250 g, for 1.5 hours. After incubation at 37°C for 6 hours, culture supernatants were replaced with fresh IMDM-10 and cells were cultured for an additional 48 hours before being used for experiments.

### Isolation of nuclear and cytoplasmic fractions and western blot analysis

Ten million OT1 T cells were washed once with PBS and rested in 0.5 mL of DPBS at 37°C for 30 minutes. Rested cells were stimulated with 1 μg/mL of anti-CD3 and 0.5 μg/mL of anti-CD28 at 37°C for 2 hours. After washing with cold PBS, harvested cells were then fractionated according to a published protocol [67]. Briefly, cells were resuspended in 40 μL of isotonic buffer (10 mM Tris pH 7.5, 2 mM MgCl_2_, 3 mM CaCl_2_, 0.3 M sucrose, 1 mM DTT) with protease and phosphatase inhibitors (Sigma) and centrifuged at 4000 rpm for 3 minutes at 4°C. After removing the supernatant, cells were resuspended in another 24 μL of isotonic buffer containing inhibitors and left on ice for 15 minutes. Triton was added to 0.1% and mixed well, and samples were centrifuged at 4000 rpm for 4 minutes at 4°C. The supernatants collected at this point constituted the cytosolic fraction. The residual pellets were resuspended with 8 μL of low-salt buffer (20 mM HEPES pH 7.9, 1.5 mM MgCl_2_, 20 mM KCl, 0.5 mM DTT, 0.2 mM EDTA, 25% Glycerol) with protease and phosphatase inhibitors, followed by adding 8 μL of high-salt buffer (20 mM HEPES pH 7.9, 1.5 mM MgCl_2_, 0.8 M KCl, 0.5 mM DTT, 0.2 mM EDTA, 25% Glycerol) with the same inhibitors. Samples were extracted on ice for at least 30 minutes and centrifuged at 13,200 rpm for 10 minutes at 4°C. The supernatants collected at this point constituted the nuclear fraction.

Whole-cell lysates were made using lysis buffer (1% Nonidet P-40, 150 mM NaCl, 50 mM Tris, pH 7.4) with freshly added protease inhibitors. Purified CD8^+^ T cells were stimulated with plate-bound anti-CD3 plus anti-CD28 for indicated times, cells lysates were made using RIPA lysis buffer with freshly added protease inhibitors. Cell lysates and nuclear and cytosolic fractions were subjected to SDS-PAGE followed by Western blot analysis using indicated antibodies (Cell Signaling Technology) as previously described [12].

### Chromatin immunoprecipitation (ChIP)

Three million/well of splenocytes from WT or DGKα^−/−^ζ*^f^*^/^*^f^* OT1 mice were activated with SIINFEKL (10 ng/mL) and IL-2 (100 U/ml) for 3 days and subsequently cultured in the presence of either IL-2 or IL-15 (10 ng/ml) for another 4 days to generate IL-2-derived effector or IL-15-derived memory cells *in vitro* [52]. Viable effector (CD44^+^CD62L^−^) and memory (CD44^+^CD62L^+^) OT1 cells were sorted for real-time RT-PCR and ChIP assay.

ChIP analysis was performed as previously described [68]. Three million sorted effector or memory cells were cross-linked with 1% formaldehyde at 25°C for 10 minutes. A final concentration of 0.125 M glycine was added to stop the reaction. Cells were then washed and lysed with NLB buffer (50 mM Tris, pH 8.1, 10 mM EDTA, 1% SDS, protease inhibitor mixture) and sonicated using a Misonics sonicator S-4000 to reduce DNA length to between 200 and 1,000 base pairs. Lysates were incubated with anti-NFκB p65 antibody (Cell Signaling, Cat 8242) at 4°C for 2 hours, and the immune complexes were subsequently precipitated with Protein A agarose beads (Invitrogen) at 4°C overnight. After being washed 5 times with LiCl wash buffer (100 mM Tris, pH 7.5, 500 mM LiCl, 1% Nonidet P-40, 1% sodium deoxycholate) and 2 times with TE (10 mM Tris pH 8.0, 1 mM EDTA), samples were resuspended in 400 μl elution buffer (1% SDS, 100 mM NaHCO3) and de-cross-linked at 65°C for 6 hours. After RNase A and proteinase K treatment, samples were extracted with phenol and chloroform. Ethanol-precipitated DNA was resuspended in Tris-EDTA buffer and analyzed by real-time RT-PCR. The primers for the analysis of the NFκB binding site in the miR-155 promoter were forward 5′-TTCCTCATGAAACCAGCTCA-3′ and reverse 5′-TTTTGACAGGGCAGAATATCG-3′. Precipitated DNA was calculated as percentages of input DNA.

### Ca2^+^ influx assay

Splenocytes at a density of 10^7^ cells/ml in loading buffer (1% FBS and 10 mM HEPES in HBSS without phenol red), were loaded with intracellular calcium indicator dye Indo-1 (2 μg/ml; Molecular Probes) in the presence of FBS and Pluronic at 30°C for 30 min. Cells were washed with loading buffer and subsequently stained with fluorochrome-conjugated Abs against CD8 and TCRVα2. Flow cytometric analysis was performed on a BD FACStar Plus cytometer. After incubated with a mixture of biotin-conjugated Abs against CD3, CD4, and CD8 (10, 5, and 5 μg/ml, respectively) at 37°C for 2-3 min, baseline fluorescence 450/510 nm ratio of the cells was measured and then streptavidin (12 μg/ml) was added to cross-link TCR and the co-receptors to trigger Ca^++^ influx. Once the ratio returned to baseline levels, thapsigargin (2 μg/ml) was added to induce maximal Ca^++^ influx.

### Statistical analysis

Data are presented as mean ± SEM, and statistical significance was determined by 2-tailed Student's *t* test. The *p* values are defined as follows: *, *p* < 0.05; **, *p* < 0.01; ***, *p* < 0.001.

## SUPPLEMENTARY MATERIAL FIGURES


