# Supplementary material for: Unexpected positive control of NFκB and miR-155 by DGKα and ζ ensures effector and memory CD8+ T cell differentiation
Source: Oncotarget. 2016 Mar 17;7(23):33744–64. doi: 10.18632/oncotarget.8164 (PMC5085116; doi:10.18632/oncotarget.8164)
Supplement: Supplementary file 1 [file oncotarget-07-33744-s001.pdf]

Unexpected positive control of NFκB and miR-155 by DGKα and ζ ensures effector and memory CD8^{+} T Cell differentiation

Supplementary Material

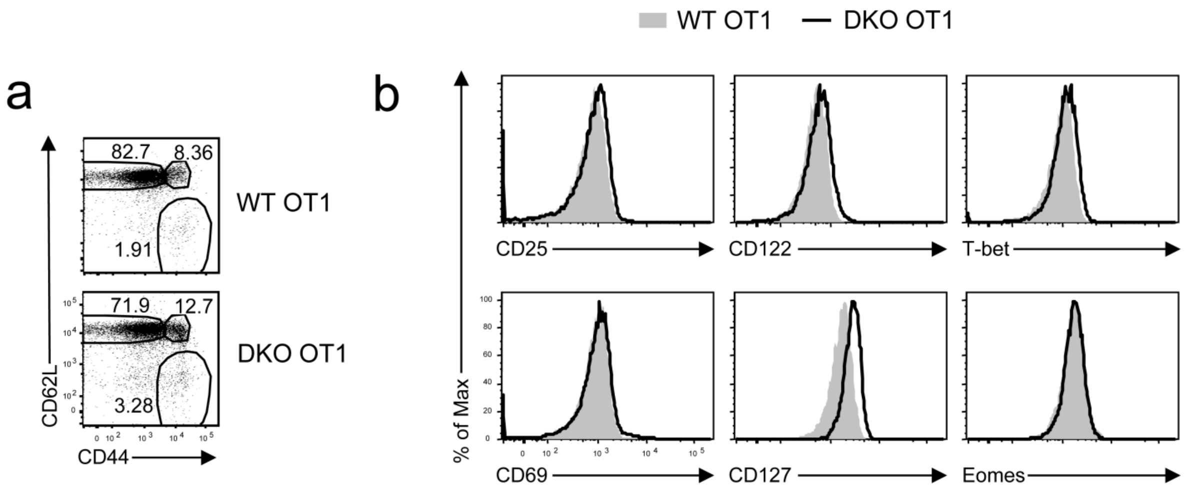

Figure S1. WT OT1 and DGKα$^{-/-}$ζ$^{f/f}$-ERCre OT1 donor mice were treated with tamoxifen. (a) CD44/CD62L staining of gated CD8^{+}TCRVα2^{+} cells from splenocytes of WT and DKO OT1 mice after tamoxifen treatment. (b) Expression of indicated molecules in gated naïve CD44^{-}CD62L^{+}CD8^{+}TCRVα2^{+} OT1 cells. Data shown are representative of three independent experiments.

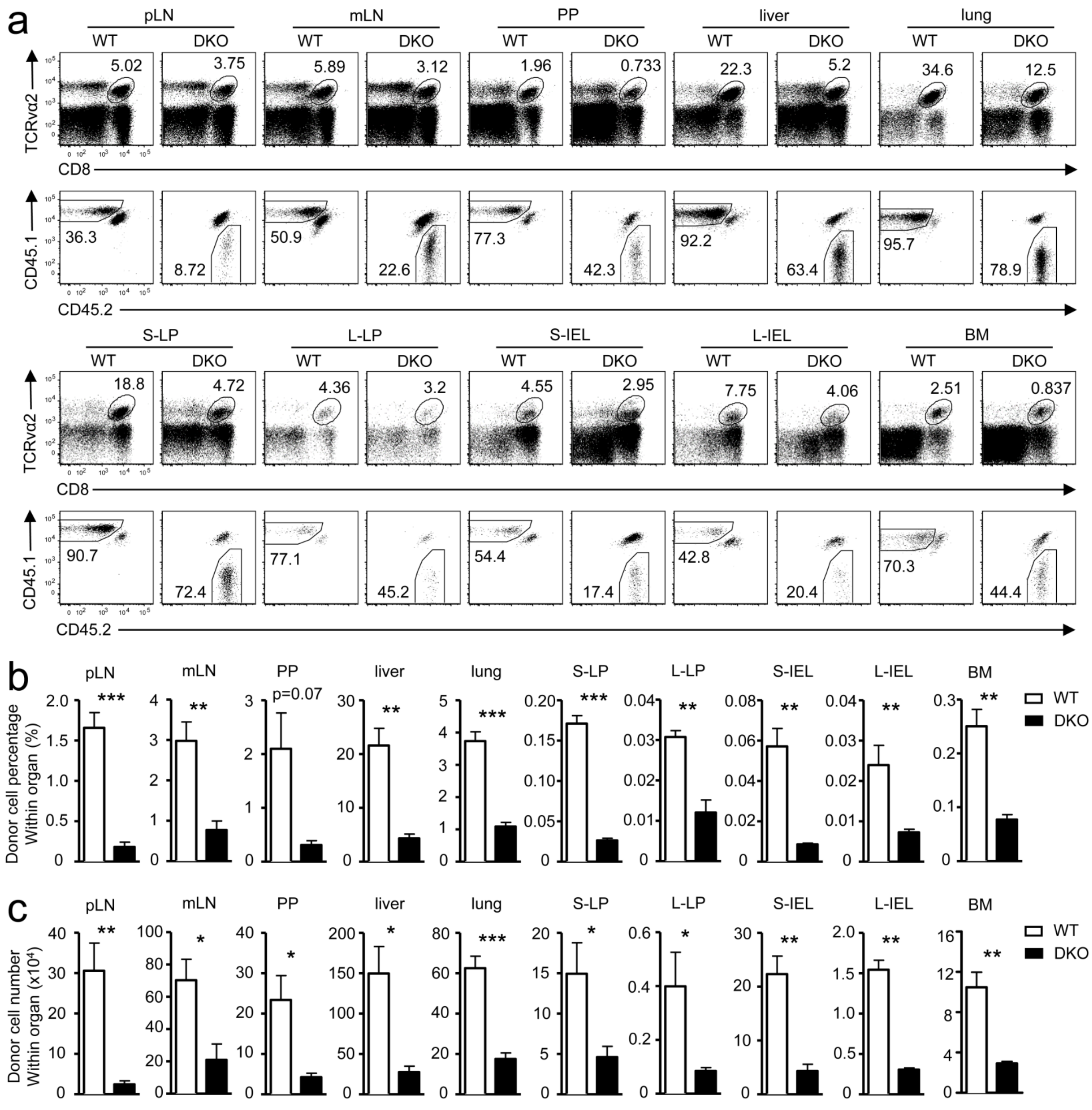

Figure S2. Deficiency of DGKα and ζ caused a global defect of CD8 effector cell expansion in lymphoid and non-lymphoid organs. (a-c) $CD45.1^{+}CD45.2^{+}$ WT recipient mice injected with $CD45.1^{+}$ WT or $CD45.2^{+}$ DKO OT 1 T cells were infected with *LM-OVA* and indicated organs were harvested and analyzed on day 7 after infection. pLN, peripheral lympho node; mLN, mesenteric lymph node; PP, peyer's patch; S-LP, small intestinal lamina propria; L-LP,

large intestinal lamina propria; S-IEL, small intestinal epithelial lymphocyte; L-IEL, large intestinal epithelial lymphocyte; BM, bone marrow. (a) Representative dot plots of indicated organs. Top panels: CD8 and TCRVα2 staining in gated live lymphoid cells. Bottom panels: Donor-derived $CD45.1^{+}CD45.2^{-}$ WT or $CD45.1^{-}CD45.2^{+}$ DKO OT1 from the gated $TCRV\alpha2^{+}CD8^{+}$ population. (b-c) Percentages (b) and absolute number (c) of OT1 T cells in indicated organs. Bars represent mean ± SEM (n=4). *, $P < 0.05$; **, $P < 0.01$; ***, $P < 0.001$ (Student's *t* test). Data shown are representative two independent experiments.

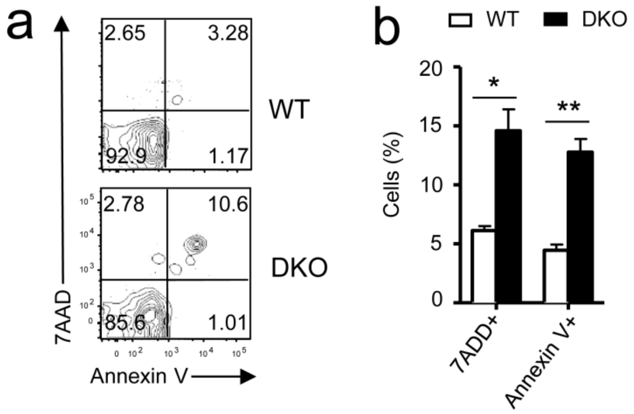

Figure S3. Increased death of DKO OT 1 memory T cells. (a–b) Frequencies of 7-AAD^{+} and Annexin V^{+} donor-derived OT1 T cells in PBLs on day 35 after *LM-OVA* infection. Data were calculated from the same experiment as in Fig. 3a.

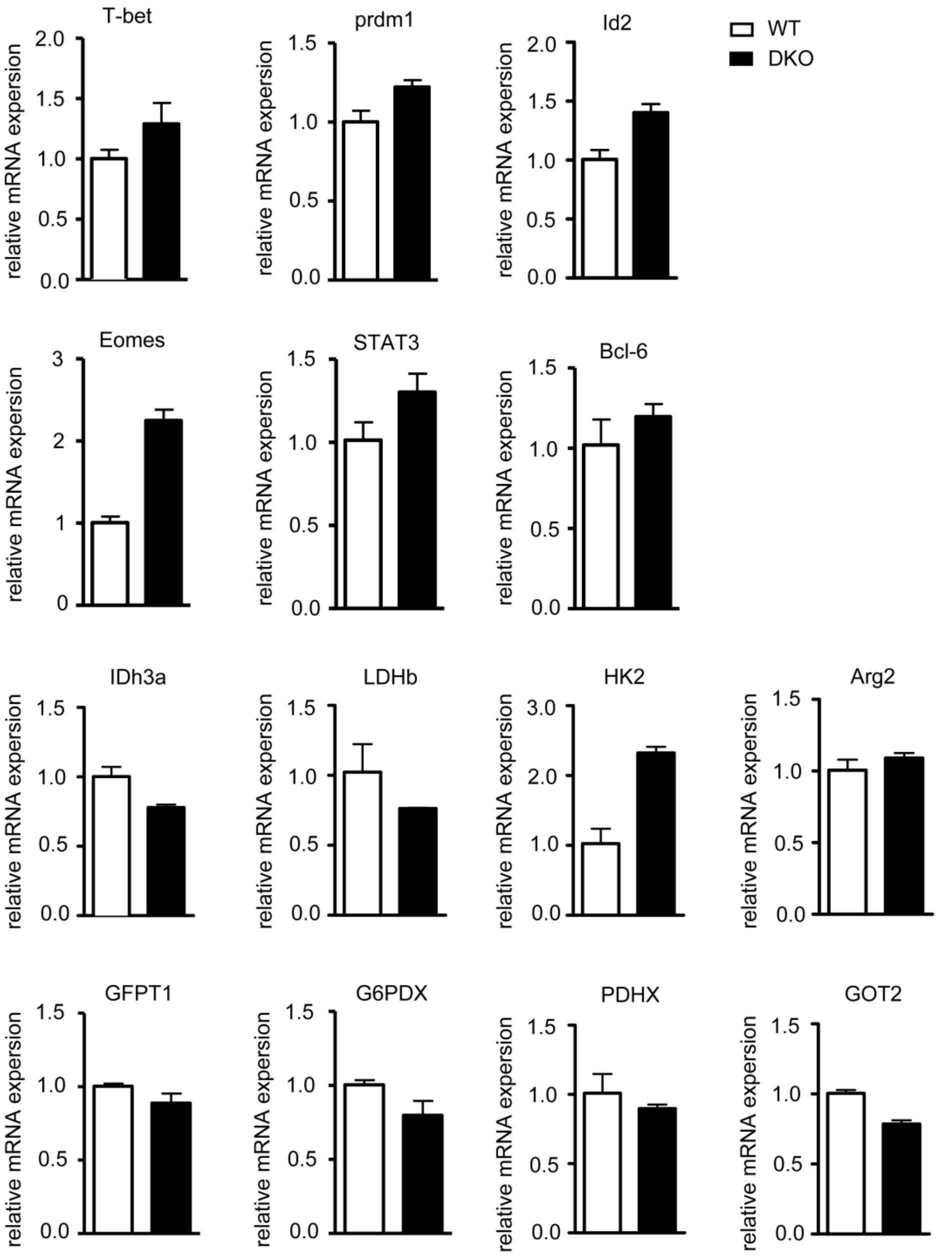

Figure S4. Expression of indicated molecules in donor-derived effector OT1 T cells sorted from splenocytes of recipients on day 7 after *LM-OVA* infection. mRAN levels of indicated molecules in sorted cells were detected by real-time qPCR. Data shown (mean ± SEM) represent values from triplicate and represent three experiments. T cell–specific T-box transcription factor

(T-bet), Eomes, Blimp-1, inhibitor of DNA binding 2 (Id2), signal transducer and activator of transcription 3 (STAT3), B cell leukemia/lymphoma 6 (Bcl-6), isocitrate dehydrogenase 3 (NAD^+) alpha (IDH3a), lactate dehydrogenase B (LDHb), arginase 2 (Arg2), glutamine-fructose-6-phosphate transaminase 1 (GFPT1), glucose-6-phosphate dehydrogenase X-linked (G6PDX), pyruvate dehydrogenase complex, component X (PDHX), hexokinase 2 (HK2), and glutamic-oxaloacetic transaminase 2 (GOT2).

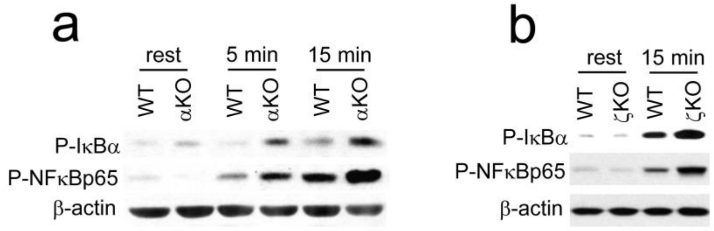

Figure S5. Elevated TCR-induced IκBα and NFκB phosphorylation in DGKα$^{-/-}$ or DGKζ$^{-/-}$ single knockout T cells. WT, DGKα$^{-/-}$, or DGKζ$^{-/-}$ CD8 T cells were rested in PBS for 30 minutes and then left unstimulated or stimulated with anti-CD3 plus anti-CD28 for 5 or 10 minutes (a, DGKα$^{-/-}$ and WT) or 15 minutes (b, DGKζ$^{-/-}$ and WT). Cell lysates were subjected to immunoblotting analysis with anti-phospho-IκBα and anti-phospho-p65 antibodies.

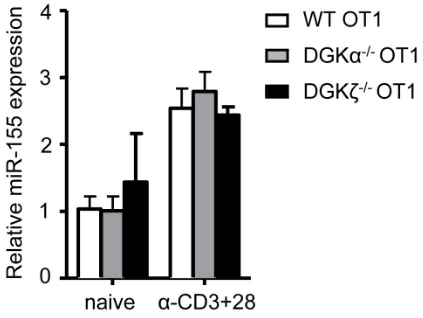

Figure S6. miR-155 expression in DGKα or DGKζ deficient OT1 T cells. Naïve OT1 T cells sorted from WT-OT1, DGKα$^{-/-}$-OT1, and DGKζ$^{-/-}$O-OT1 mice were either directly used to make RNA or after stimulation with plate-bound anti-CD3 and CD28 for 18 hours. miR-155 levels in these samples were measured by real-time qPCR.
